# Environmentally friendly recycling system for epoxy resin with dynamic covalent bonding

**DOI:** 10.1080/14686996.2021.1897480

**Published:** 2021-07-15

**Authors:** Hsing-Ying Tsai, Takehiro Fujita, Siqian Wang, Masanobu Naito

**Affiliations:** a Data-driven Polymer Design Group, Research and Services Division of Materials Data and Integrated System (Madis), National Institute for Materials Science (NIMS), Tsukuba, Japan; b Program in Materials Science and Engineering, Graduate School of Pure and Applied Sciences, University of Tsukuba, Tsukuba, Japan

**Keywords:** Epoxy resin, aromatic disulfide bonding, degradation, glutathione, recycling, 10 Engineering and Structural materials; 301 Chemical syntheses / processing

## Abstract

Recycling of epoxy resin and its composites is extremely difficult due to its thermoset nature. In this study, we proposed the environmentally-friendly recycling system of epoxy resin with dynamic covalent bonding in the assistance of cysteine-containing tripeptide, so-called glutathione. The glutathione attached on the epoxy resin and resulted in the cleavage of dynamic disulfide bonds of epoxy resin through thiol-disulfide exchange reaction between the thiol group of glutathione and disulfide bonding of epoxy resin, followed by the scission of epoxy networks. Therefore, the degraded epoxy residue was dissolved into chloroform. Finally, this resulting product could be reused as reagent for preparation the new epoxy materials with approximately 90% of initial mechanical strength via regeneration of disulfide bonding through heating. This work demonstrated the different aspect to understand the decomposition and recycling of thermosetting networks and the wide application under more environmentally friendly condition.

## GRAPHICAL ABSTRACT

**Figure:**
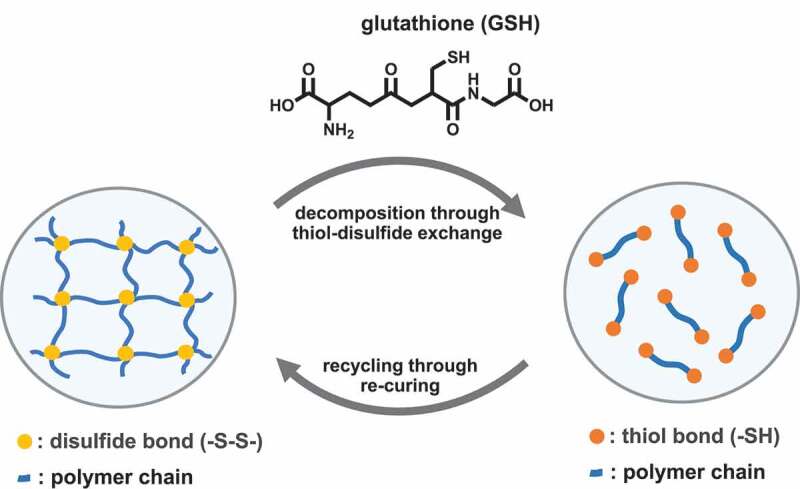

## Introduction

Epoxy resin is one of the most common polymeric materials, which has been used in wide ranges of applications, such as coating, paint, primer, adhesive, and composites. In general, epoxy resin exhibits excellent thermal, mechanical, and chemical resistant properties due to its crosslinked polymer network [1–6]. However, such the crosslinked network polymers often adapt thermosetting nature, leading to less recyclability and reworkability. Therefore, the epoxy resin and its composites are usually disposed by landfilling and incineration, which cause significant negative impacts on whole ecosystems. In particular, the epoxy resins used in our daily life is considered to be one of the sources of microplastics [7–10]. Thus, it is highly desired to develop the recyclable and reworkable thermoset epoxy resin to reduce the waste accumulation. To address this social demand, dynamic covalent bonding which is a reversible chemical network under specific stimuli have been introduced into the thermoset epoxy resin. This new class of polymers are known as a vitrimer, which can change their network topology by thermally activated bond-exchange reaction, while still remaining many of the beneficial properties of thermosets [11–15]. Among them, it was reported that a thermoset epoxy resin with disulfide bonding (termed as **ERD** herein) showed excellent vitrimer nature under the external stimuli such as heat or UV-irradiation [16–21]. For example, our group reported that the epoxy resins incorporating aromatic disulfide bonds demonstrated improving adhesive properties with increasing temperature along with vitrimer nature [21].

Disulfide bonding plays an important role not only as dynamic covalent bond, but also in ERD recycling. Recently, Alaitz Ruiz de Luzuriaga and co-workers demonstrated that the ERD can be used as a recyclable matrix for carbon fibre reinforced plastics (CFRP), in which 4,4ʹ-dithiodianiline (DTDA) was used as the disulfide-containing amine hardener. When the CFRP with ERD matrix was soaked into 2-mercaptoethanol (2-ME)/dimethylformamide (DMF) solution, ERD completely dissolved within 24 hours at room temperature, and the carbon fibre was recovered without contamination of ERD residue. Here, thiol-disulfide exchange reaction is supposed to occur among the disulfide bonding (-S-S-) in ERD and the thiol (-SH) in 2-ME as a solvent [16,19,20]. However, the decomposed epoxy residue has not been further considered as a recycled resource. In addition, 2-ME is of concern as a toxic chemical. Therefore, it may not be suit for practical usage from viewpoint of environmental concern. In other words, if we could recycle epoxy resin with environmentally friendly waterborne reagent, it would greatly reduce the environmental impact and accelerate the reuse of thermosetting epoxy resins.

In this paper, we proposed an environmentally friendly recycling system of ERD that mimics the drug metabolism in living organisms. Herein, we focused on glutathione (γ-glutamyl-cysteinyl-glycine, GSH), cysteine-containing tripeptide as a waterborne thiol-containing reductant. GSH is renowned as a natural antioxidant in organism. GSH has an antioxidant effect by reducing reactive oxygen species and peroxides using its own thiol group, and a detoxification effect by S-S bonding (glutathione conjugation) to the thiol group of cysteine residues of various poisons and drugs, thereby playing a role in protecting against cell injury and death, canceration, and aging [22–25]. Here, we expected that waterborne GSH enables us to mediate cleavages of the disulfide bonding in ERD by thiol-disulfide exchange reaction. Consequently, ERD was disassembled into small oligomers under the mediation of GSH at room temperature in water/organic solvent binary system. The resulting liquid epoxy residue as decomposition was curable upon heating at 180°C for 6 hours by regeneration of disulfide bonding among ERD residues. The obtained solid ERD exhibited 90% of storage modulus compared to the virgin epoxy resin, and the value was almost identical even after several recycling cycles. Finally, CFRP structure was fabricated with ERD (CFRP-ERD). The CFRP-ERD was decomposed using GSH aqueous solution and the carbon fibers were successfully recovered without contamination of ERD. It was further demonstrated that the liquid ERD residue could be cured to form ERD again.

## Results and discussions

### Synthesis

**Scheme 1.**
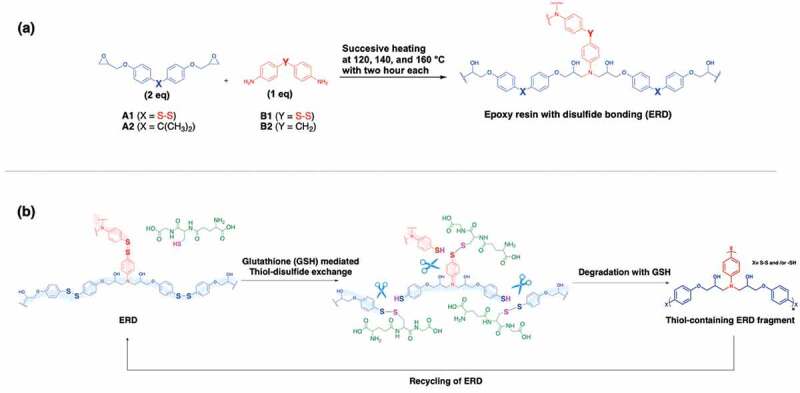
(a) Chemical structure of compounds used in this study, and (b) schematic representation of GSH mediated recycling system of ERD

ERD was prepared with bis(4-glycidyloxyphenyl) disulfide (BGPDS, A1) and 4,4ʹ-dithiodianiline (DTDA, B1) as an epoxy monomer and diamine hardener, respectively (Scheme 1). In order to clarify the effect of thiol-disulfide exchange reaction, diglycidyl ether of bisphenol A (DGEBA, A2) and diaminodiphenyl methane (DDM, B2) were employed as analogues of BGPDS and DTDA without disulfide bonding, respectively. The chemical reactivities of the aromatic disulfide in the epoxy monomer and amine hardener were assumed to be almost identical. ERD were prepared by combination of the epoxy monomer (either A1 or A2) and the diamine hardener (either B1 or B2). The detailed combination was summarized in Table 1. As a precuring process, the mixture of epoxy monomer and diamine hardener was stirred at 90°C for 30 mins in stoichiometric molar ratio (2: 1). The precured mixture was transferred onto the Teflon mould, and successively cured at 120, 140 and 160°C with two hour each. After cooling, ERD was obtained as a brown solid material.

**Table 1.** Epoxy resin with disulfide bonding (ERD) used in this study. ERD-C25 was used as a model without disulfide bonding

		Molar ratio of diamine hardener (B1/B2)				
		100/0	75/25	50/50	25/75	0/100
Molar ratio of epoxy monomer (A1/A2)	**100/0**	C1	C2	C3	C4	C5
	**75/25**	C6	C7	C8	C9	C10
	**50/50**	C11	C12	C13	C14	C15
	**25/75**	C16	C17	C18	C19	C20
	**0/100**	C21	C22	C23	C24	C25

**Figure 1.**
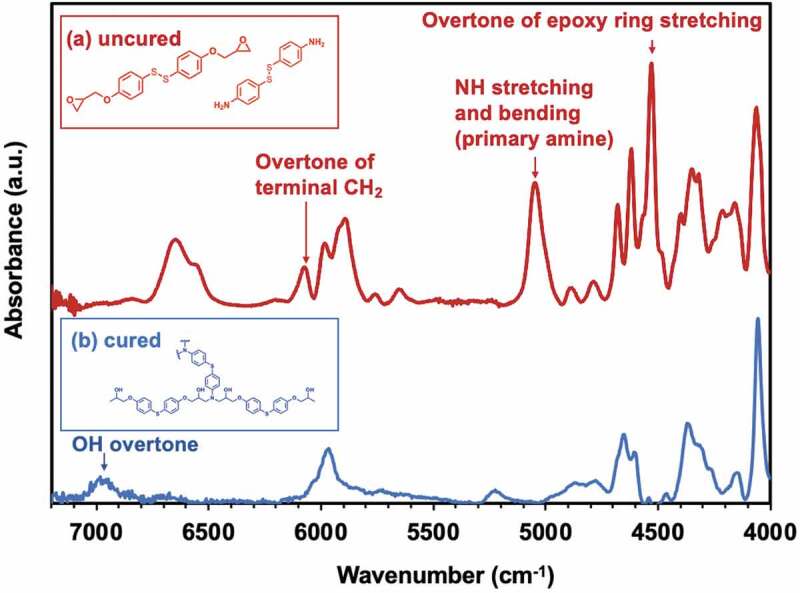
FT-nIR spectra of uncured (red) and cured ERD (blue)

Note that **C25** was used as a control for not containing disulfide bond, whereas the others have some amount of disulfide bonds. In order to monitor curing process, Fourier transform near-infrared spectroscopy (FT-nIR) was performed. Figure 1 is a typical example of (a) uncured and cured ERD with **C1** combination. In the nIR region from 7200–4000 cm^−1^, well defined bands related with the epoxy and primary amine are observable as the combination band of the second overtone of the epoxy ring stretching with the fundamental C-H stretching (ca. 4530 cm^−1^) and the combined band of NH stretching and bending (ca. 5000–5100 cm^−1^). Thus, the epoxy monomer decreased upon curing process, resulting in decrease of the band of the fundamental C-H stretching and the weak overtone of terminal CH_2_ from ca. 4530 cm^−1^ and ca. 6060 cm^−1^, respectively. The primary amine combination band at ca. 5000 cm^−1^ also decreased. On the other hand, the band of O-H overtones at ca. 7000 cm^−1^ increased as a consequence of the oxirane ring opening reaction.

### Degradation of ERD with GSH

Prior to demonstrate degradation of ERD with GSH, we performed degradation experiment using a disulfide-containing small molecule in a water/organic binary system. Here, DTDA was chosen as a model with disulfide containing molecule. DTDA and GSH was dissolved in deuterated chloroform (CDCl_3_) (250 mM) and deuterated water (D_2_O) (250 mM), respectively. The solution was mixed with 1:1 (v/v) and kept in dark covered by aluminium foil to avoid unexpected photo-induced reaction.

**Figure 2.**
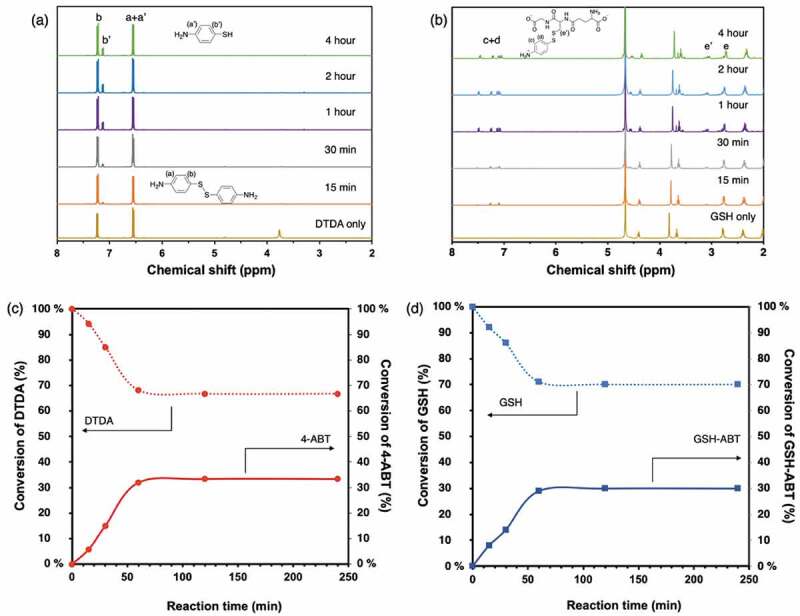
NMR spectra of (a) solvent phase and (b) water phase for GSH-assisted thiol-disulfide exchange reaction of model compounds at different reaction time. Time evolution of product by reaction of DTDA with GSH in (c) CDCl_3_ and (d) D_2_O. Amount of the product was monitored by ^1^H-NMR at 7.22, 7.12, 2.8, and 3.12 ppm DTDA, 4-ABT, GSH, and GSH-ABT, respectively

**Figure 3.**
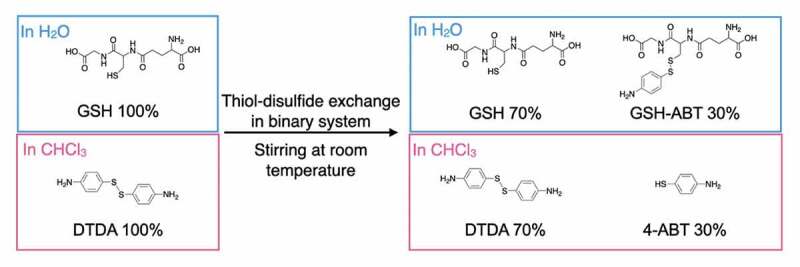
Chemical reaction balance for CDCl_3_ (CHCl_3_) and D_2_O (H_2_O) phases

The reaction mixture was quantitatively evaluated with ^1^H-NMR spectroscopy (Figure 2 and Figure S2). Figure 2(a,b) showed change in ^1^H-NMR spectra of CDCl_3_ and D_2_O phase over time, respectively. In CDCl_3_ phase, ^1^H-NMR signals of an aromatic ring in DTDA (a) and (b) appeared at 6.55 and 7.22 ppm, and ^1^H-NMR signal at 7.12 ppm was assigned as an aromatic ring of 4-aminobenzenethiol (4-ABT) (b’), which supposes to be a reductant of DTDA (Figure 2(a)). In addition, no peak was observed in the range of 2–3 ppm, suggesting that the GSH and the reactants with GSH did not exist in CDCl_3_ phase. On the other hand, in D_2_O phase, new ^1^H-NMR signal (e’) appeared at 3.12 ppm, suggesting formation of S-S bond between GSH and 4-ABT (Figure 2(b)). In addition, the ^1^H-NMR peaks (c) and (d) appeared at 7.15 and 7.5 ppm and the intensity was increased simultaneously with peak (e’), indicating that the generation of water-soluble reactant GSH-ABT through exchange reaction between DTDA and GSH. Also, considering that there was no significant change in the intensity of peaks at 7.25 and 7.5 ppm, these two peaks indicated the that slight amount of DTDA was dissolved into water phase. Compared to the diphenyl disulfide as stated in Figure S2, the solubility of DTDA in water was enhanced, promoting the exchange reaction between thiol bonding in GSH and disulfide bonding in DTDA. Next, the time evolution of the composition in CDCl_3_/D_2_O was further evaluated. Figure 2(c) shows a rate of change of DTDA and 4-aminobenzenthiol calculated from the peak areas of ^1^H-NMR signal (b) and signal (b’) in CDCl_3_, respectively. Consequently, DTDA was immediately decreased with time and reached equilibrium at 60 min. Correspondingly, 4-ABT began to generate immediately after stirring and reached equilibrium at 60 min in a symmetrical manner to DTDA. Similarly, Figure 2(d) shows the time evolution of decrease in GSH and increase in the water-soluble reactant of GSH and 4-ABT in D_2_O phase. As in CDCl_3_ phase, increase in GSH and decrease in the reactant of GSH/4-ABT tended to change symmetrically, reaching equilibrium in approximately 60 mins. More importantly, the molar rate of increased and decreased compositions was kept at 30 mol% for both the CDCl_3_ and D_2_O phase. From these results, the chemical reaction balance in each of CDCl_3_ and D_2_O phases was proposed in Figure 3. Consequently, the thiol-disulfide exchange reaction between DTDA and GSH underwent at the interface of CDCl_3_ and D_2_O, resulting in generation of the water-soluble reactant of GSH and 4-ABT (GSH-ABT), and unreacted 4-ABT remained dissolved in CDCl_3_.

**Figure 4.**
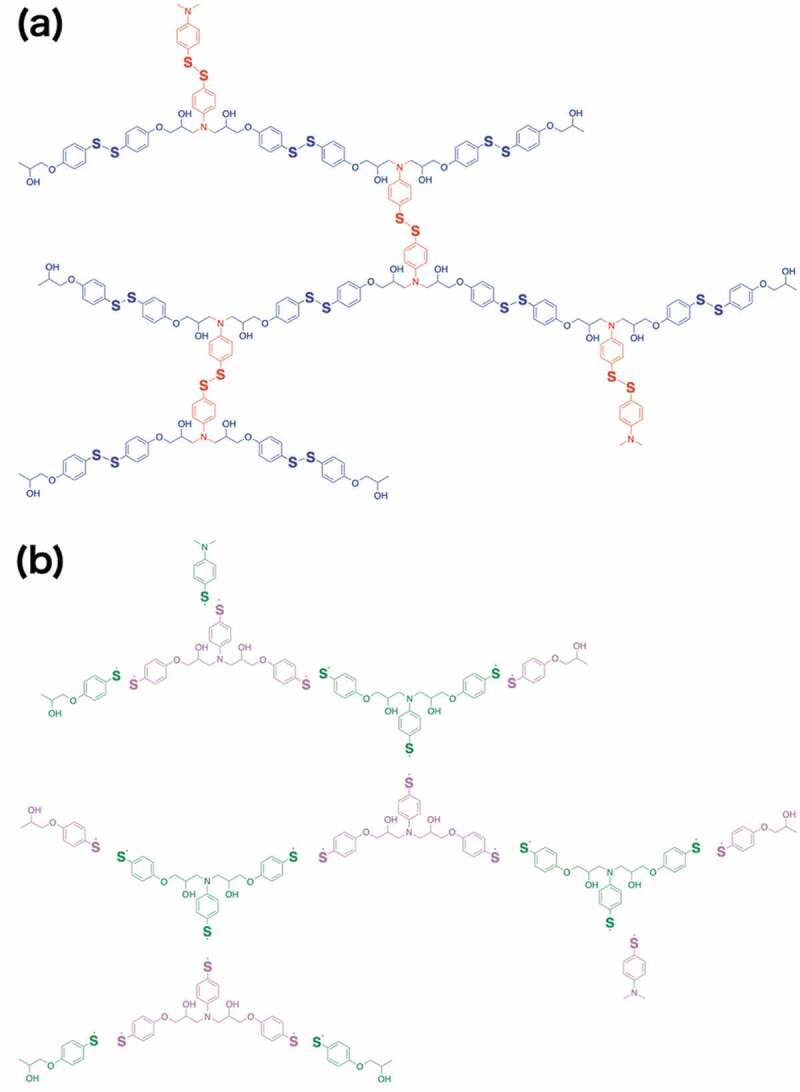
Schematic illustration of repeating unit of ERD with (a) cross-link point of amine bonding and (b) degradable disulfide bonding

Since it was confirmed that the thiol-disulfide exchange reaction proceeds in CHCl_3_/water binary system with GSH mediation, the disassembly test of ERD was conducted with the same binary condition. First, ERD was ground by a ball milling method at a shaking frequency of 25 Hz for 1 hour. The obtained ERD powder was suspended in CHCl_3_, and GSH aqueous solution (20 mM) was added. Here, tributylphosphine (TBP) (10 mol%) of ERD was used to accelerate the thiol-disulfide exchange reaction [26]. The ^1^H-NMR spectra of small molecular model with TBP presented in Figure S2 proved that the products generated through thiol-disulfide exchange reaction were identical as the one without TBP in Figure S3. This binary solution was vigorously stirred at room temperature. After certain period of time, the results were divided into two types of features: those in which the ERD was completely dissolved in CHCl_3_ phase, resulting in a yellow solution, and those in which precipitation occurred. The result of decomposition of ERDs was summarized in terms of composition ratio of the epoxy monomer (either A1 or A2) and the diamine hardener (either B1 or B2) (Table 2). Along with this, molar equivalents of disulfide bonds in each repeating unit of ERD by various combinations of BGPDS/DGEBA and DTDA/DDM in various proportions were summarized. In Table 2 (a), the compositions in which ERD was completely dissolved were marked in red. It should be noted that the amine hardener (DTDA or DDM) can react with two equivalents of the epoxy monomer (BGPDS or DGEBA), which suggest that the disulfide bonds can be introduced up to three equivalents of the repeating units of ERD (Figure 4). From completely dissolved parts, the viscous yellow liquid was obtained after removing CHCl_3_ solvent under vacuum.

**Table 2.** Photograph of the resultant of decomposed ERD in CHCl_3_/water binary system with GSH. Compositions in which ERD was completely dissolved were marked in pink

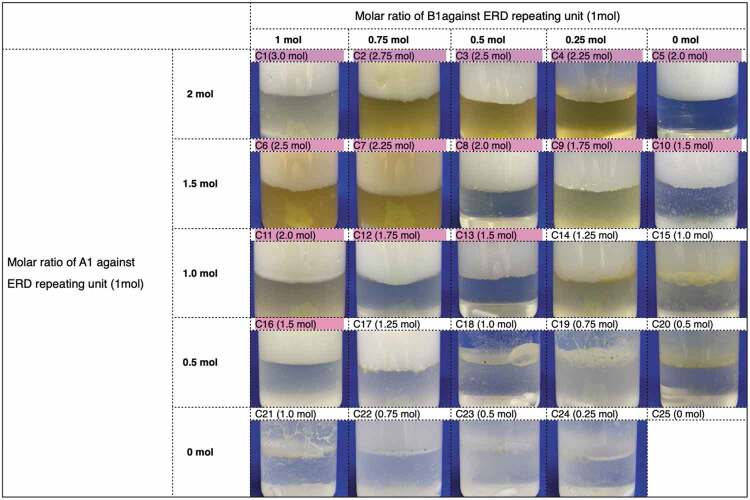

**Figure 5.**
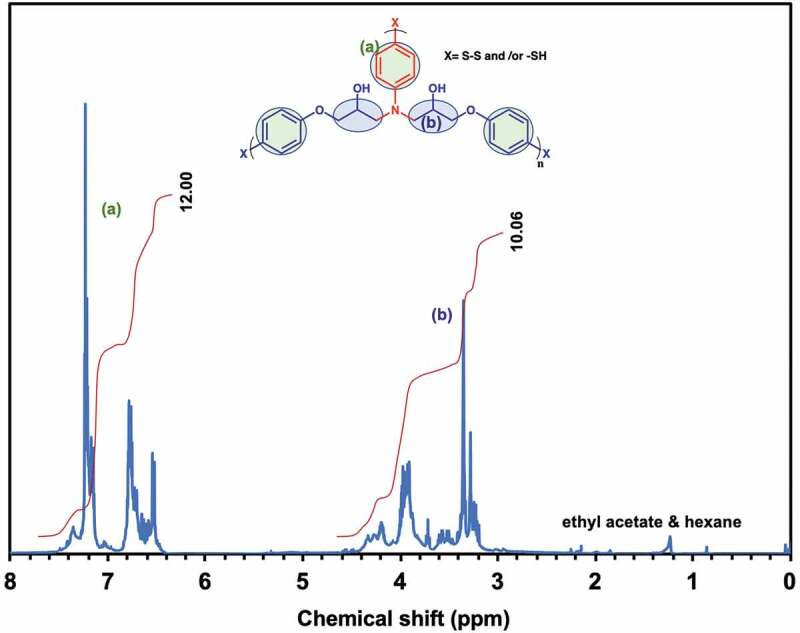
NMR spectrum of decomposed ERD-C1

**Figure 6.**
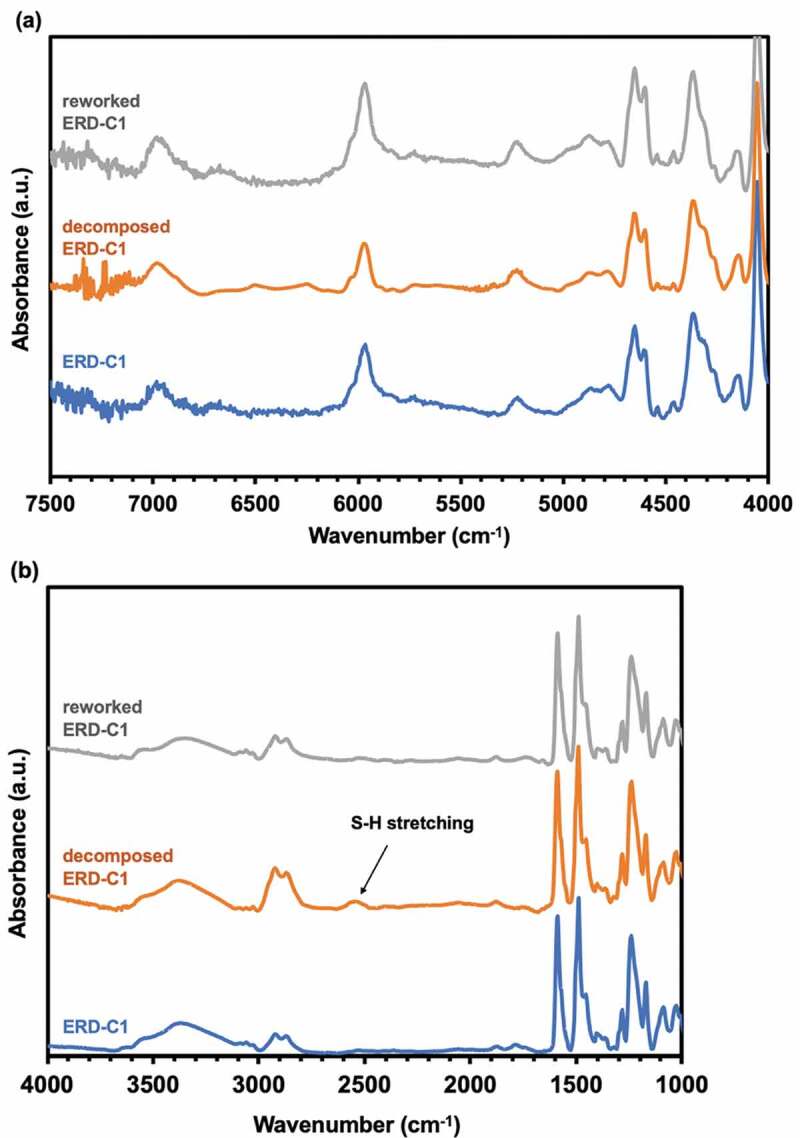
(a) FT-nIR and (b) FT-mIR spectra of ERD-C1, decomposed ERD-C1, and reworked ERD-C1 after curing

To identify the chemical structure of these residues, ^1^H-NMR and FT-nIR spectroscopy was conducted in ERD-C1, respectively. Figure 5 presented the ^1^H-NMR spectrum of CDCl_3_ phase after removing the tributylphosphine, suggesting the pure degraded ERD-C1. In this spectrum, signals of aromatic ring in decomposed ERD were detected at the range from 6.5 to 7.5 ppm. Also, the peaks between 3.0 and 4.0 ppm were related to the alkyl protons generated from the ring-opening reaction of epoxide groups. In addition, no peak was detected in the range from 2.0 to 3.0 ppm, which proved that the GSH and the exchange product did not exist in CDCl_3_ phase. Also, the tributylphosphine was entirely removed since there was no signals identified at the range from 1.0 to 1.5 ppm. Therefore, this result showed that the ERD disassembled into soluble oligomers as assumption in Scheme 1(b) but remained the epoxy structure. Furthermore, FT-IR spectrum of the decomposed soluble portion of ERD-C1 in CHCl_3_ phase was as shown in Figure 6. Figure 6(a) presented that the spectrum of this residue was almost identical to that of ERD in the nIR region from 4000 cm^−1^ to 7500 cm^−1^. This implies that the liquid residue retains the epoxy structure of ERD. On the other hand, a new thiol-derived absorption band appeared at 2550 cm^−1^ (Figure 6(b)). This suggests that this fragment contains the thiol group by the reduction of the disulfide group of ERD by GSH.

**Figure 7.**
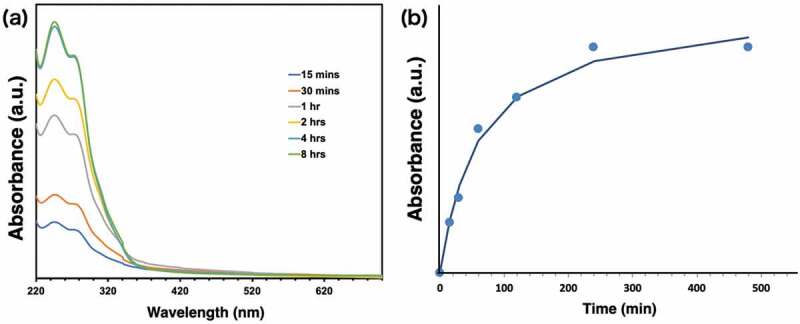
(a) Time-dependent UV-vis spectra for decomposed ERD-C1 in CHCl_3_ phase, and (b) time course of UV spectra of decomposed ERD-C1 monitored at 254 nm

Here, the solubility of ERD can be discussed in terms of the stoichiometric ratio of the disulfide group and the repeating units of ERD (Table 2). ERD completely dissolved in CHCl_3_ when the disulfide group was more than 1.5 equivalents to the repeating unit of ERD, in which ERD was decomposed into dimeric or monomeric units. On the other hand, If the stoichiometric ratio of the disulfide groups was less than 1.5, most of the repeating unit would be trimeric or higher, resulting in precipitates. To further investigate degradation tendency of ERD, UV-vis spectra in CHCl_3_ phase were measured at 254 nm with time (Figure 7). When the suspension of ERD-C1 in CHCl_3_ was mixed with GSH/aqueous solution, UV absorption at 245 nm immediately increased and followed the saturation curve. After four hours, it reached a plateau region with a constant value, suggesting that ERD-C1 completely dissolved in CHCl_3_ phase. The degradation ability of ERD was clearly correlated with the number of disulfide bonds in epoxy resin.

### Reworking test

In general, amine-curing epoxy resins are regarded as network structures with amine bonds as cross-linking points, but in the case of ERD, it can also be regarded as dynamic covalent network polymers with disulfide bonds as the repeating units (Figure 4(b)). Therefore, it was assumed that the structure and physical properties of ERD as the original amine-cured epoxy resin would not change even if ERD was degraded and regenerated by formation of disulfide bonds.

**Figure 8.**
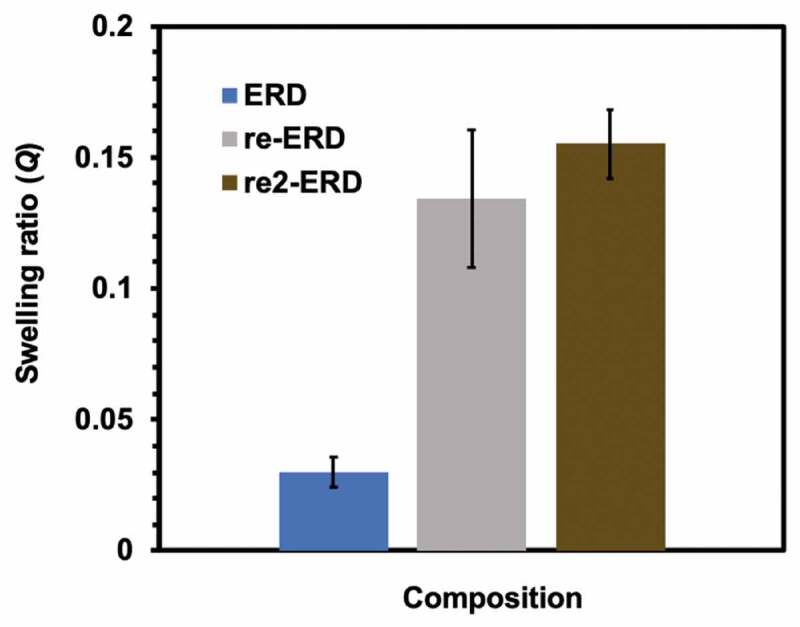
Swelling ratio for ERD, 1st-time reworked ERD, and 2nd-time reworked ERD in toluene at room temperature for 72 hours

The degraded epoxy residue was poured into Teflon mould, and heated at 180 °C for 6 hours. As a result, the liquid residue turned to dark brown solid. FT-nIR measurement clearly revealed that the recycled ERD was cured by formation of disulfide bonding. Thus, the decomposed yellow liquid contained thiol group whereas the peak at 2550 cm^−1^ corresponding to thiol group vanished after curing (Figure 6(b)). In order to evaluate the thermal and mechanical property of disulfide-contained epoxy resin before and after recycling, the dynamic mechanical analysis (DMA) was performed (Table 3 and Table S3). For mechanical property, the storage modulus of the original ERD was 1.8 GPa, presenting the comparable storage modulus as the conventional epoxy resin without disulfide bonding (1.88 GPa) [18,19,21]. After recycling, the storage modulus of reworked ERD maintained approximately 90% of initial value at room temperature. On the other hand, glass transition temperature (*T*_g_) was greatly decreased from 131°C to 82°C due to the incomplete network reconnection. The swelling test for pristine and recycled was also performed to evaluate the crosslinking density before and after degradation and recycling. In Figure 8, the swelling ratio after 72-hour immersion in toluene at room temperature for initial cured sample was around 3%, while the swelling ratio for recycled epoxy resin was increased to 13–16%.

**Table 3.** Thermal and mechanical properties for ERD and reworked ERD

Properties	ERD	Recycled ERD (1st time)	Recycled ERD (2nd time)
Glass transition temperature (*T*_g_) (°C) (DSC)	130.9	82.7	81.7
Storage modulus (*E*’) at 25 °C (GPa) (DMA)	1.8	1.6	1.7
Storage modulus (*E*’) (Rubbery state) (at *T*_g_ +30 °C) (MPa) (DMA)	14.3	49.9	26.8

**Figure 9.**
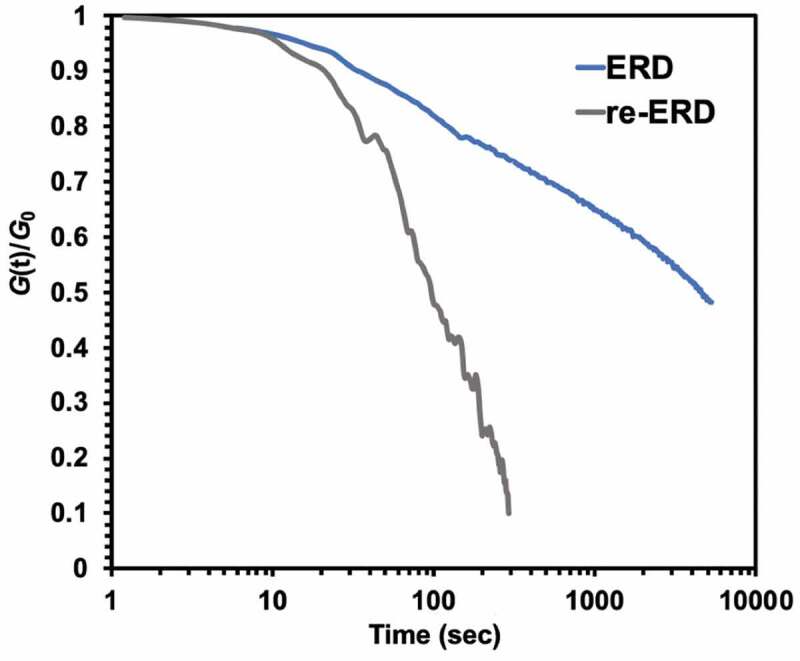
Stress relaxation for ERD and reworked ERD at 130 °C

This result proved that the crosslinking density after recycling could not be completely resumed to original structure, causing the lower glass transition temperature and decay of mechanical strength at room temperature (from 1.8 GPa to 1.6 GPa) but higher storage modulus in rubbery state (from 14.3 MPa to 49.9 MPa). The time-dependent relaxation examined by DMA was the other key characteristic for dynamic disulfide network to assess heat-induced malleability and ductility. Figure 9 showed the normalized stress relaxation of pristine and recycled epoxy resin at 130 °C. Based on Maxwell model equation, the relaxation time was defined as the time required to release 63% of initial stress. The relaxation time of recycled network was 152 seconds at 130 °C, exhibiting more rapid stress release than original one. The result demonstrated that recycled network showed stress relaxation above *T*_g_ as general dynamic network attributed to reformation of dynamic disulfide bonds. Besides, due to relatively low *T*_g_ of recycled epoxy resin, the segmental chain motion could be occurred in lower temperature, resulting in faster exchange reaction and then stress relaxation phenomenon.

### Recycling of CFRP

CFRP structure is a structural material that is attracting attention in fields such as aircraft and automobiles where weight reduction and creep resistance are required. In general, thermosetting resins such as epoxy are used as the matrix resin of CFRP structure, making it difficult to rework and reuse, and the problem of waste disposal has become apparent. Therefore, research and development of CFRP structure using thermoplastic resins such as polypropylene is also underway. However, thermoplastic resins generally have lower mechanical strength than thermoset resins. In addition, from the viewpoint of recycling, it is difficult to completely separate the carbon fiber from the resin even if thermoplastic resin is used, and the establishment of a more optimal recycling system is eagerly awaited.

**Figure 10.**
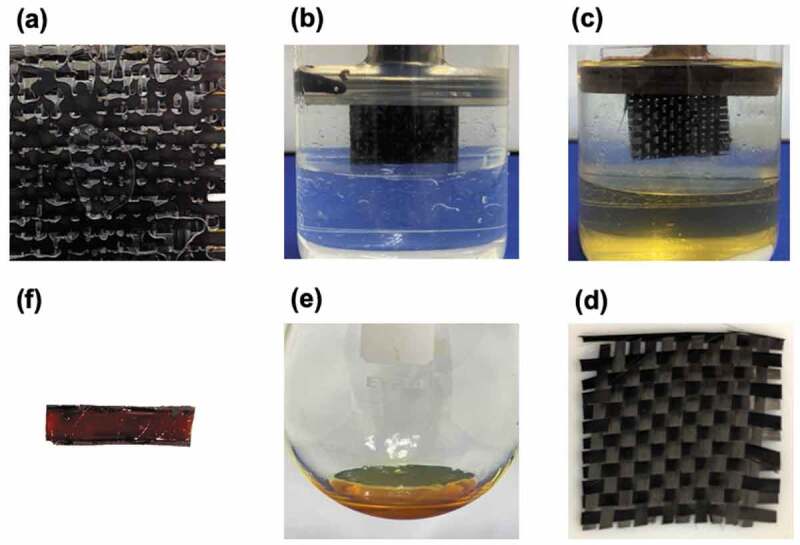
Demonstration of recycling procedure for carbon fiber reinforced composite (CFRP) (a) CFRP with ERD-C1, (b) Decomposition test of CFRP-ERD with GSH, (c) after 24 hours, (d) recovered carbon fiber, (e) ERD residue recovered from CHCl_3_ phase, and (f) reworked ERD cured at 180°C for 6 hours

Finally, recycling system of CFRP structure with ERD matrix was proposed. The cured CFRP structure was fixed by clip in binary solution (Figure 10(a,b). By intensely stirring into one homogeneous mixture at ambient condition for 24 hours, ERD as matrix of CFRP structure was decomposed into CHCl_3_ phase (Figure 10(c)). The carbon fiber was completely recovered via washing by water and acetone and then drying at 100 °C after matrix dissolution (Figure 10(d)). On the other hand, the decomposed epoxy residue dissolved in chloroform solution was obtained through solvent evaporation (Figure 10(e)). The epoxy residue could be simply transferred to resin network by formation of disulfide bonding (Figure 10(f)).

## Conclusion

In summary, the peptide-assisted reworking and recycling system for epoxy resin with aromatic disulfide bonds was presented in this paper. First, by introducing the dynamic S-S bonds, we obtained the recyclable and reworkable epoxy resin with disulfide bonding. Then, dynamic disulfide bonds in epoxy resin could be broken and exchanged with S-H bonds of glutathione via thiol-disulfide exchange reaction, leading to the dissolution of degraded epoxy residue in chloroform and exchange product would be distributed in aqueous phase due to the existence of hydrophilic peptide groups in glutathione. Finally, the epoxy residue with SH bonds was heated and reformed to the disulfide-contained epoxy networks. The resulting bulk recycled resin maintained 90% of mechanical strength of pristine epoxy resin, presenting the potential on different application. This approach could be practically used in carbon fiber reinforced composites, which may expand the range of reusing both embedded objects and matrix resin. Through this strategy, the research of reworking and recycling may be worthy further discussed in the future. Eventually, it may provide a practical solution to solve the microplastics, which is a growing concern around the world.
